# Early career researchers in health policy and systems research: insights from freelancers in a non-profit organization in the Philippines

**DOI:** 10.1186/s12961-024-01142-6

**Published:** 2024-04-29

**Authors:** Reneepearl Kim Sales, Marion Abilene Navarro

**Affiliations:** 1 Alliance for Improving Health Outcomes, Veria 1 Building, 62 West Avenue, West Triangle, 1104 Quezon City, Philippines; 2 Freelance Early Career Researcher, Manila, Philippines

**Keywords:** Public health research, Health policy and systems research, Freelance researchers, Early career researchers, Public health, Philippines, Career development, Research culture

## Abstract

**Background:**

The freelance economy has seen rapid growth worldwide in recent years and the Philippines is not an exception. Freelance workers are becoming increasingly common in healthcare and research. Early career researchers carry out most of scientific research and can play a critical role in advancing public health by bringing new perspectives and diversity to the field. Existing literature has mostly focused on the experiences of early career researchers in an institutional academic setting. This study aimed to understand the experiences of freelance early career researchers in the health policy and systems space in the Philippines.

**Methods:**

This qualitative study collected data from 18 to 22 March 2022 through virtual interview and focus group discussions. Themes and codes were created based on the topic guide developed. New themes and codes were generated as they emerged. Two researchers coded the data using both a priori and emergent codes. Any coding conflicts were resolved through discussions until intercoder agreement was reached. Interpretation and conclusions from the data were developed by 2 researchers with consideration for its context and relationship between themes.

**Results:**

Fifteen current and former freelance researchers participated in the study. Most are female, under 35 years old, and with an undergraduate degree as the highest educational attainment. The findings highlight insights and challenges faced by early career researchers in aspects of: (1) work arrangement, (2) tasks, (3) expectations from senior researchers, (4) development in the health policy and systems field, (5) relationship with peers, and (6) motivations for continuing to work as a freelance health policy and systems researcher.

**Conclusion:**

This study reveals the challenges freelance early career researchers face, highlighting the need for enhanced support and recognition amidst rapidly evolving workforce demands and complex health dilemmas. Recommendations include structured mentorship, professional development, innovative funding models, and the establishment of a supportive network. Advocacy for policies ensuring freelancer inclusion in the economy and policy-making is crucial. Future research should investigate their experiences further, including their roles, transitions, and the impacts of funding trends, to foster their development and integration into public health research and policy.

**Supplementary Information:**

The online version contains supplementary material available at 10.1186/s12961-024-01142-6.

## Introduction

The freelance economy has seen rapid growth worldwide in recent years, and the Philippines is not an exception. The country ranked 6th among the fastest growing markets for gig economy in the 2019 Gig Economy Index [1]. Young workers dominate the freelance landscape in the country, with 67% of freelancers being under the age of 35 years old [1, 2]. While experience is not the only determinant for pay rates of freelancers, those with only up to 2 years of experience earn less than Php 30,000.00 per month (approximately USD 545.00) [3].

Despite the rising number of freelancers in the country based on global reports, the Philippine Department of Labor and Employment continues to lack baseline data on freelance workers due to challenges of registering individuals that work in non-traditional settings [4]. Labor laws for freelancers are often inadequate in low- and middle-income countries (LMICs), leaving them vulnerable and without safety nets. A lack of baseline data often contributes to policy inaction to protect freelance workers. Despite sparse data in the Philippines, Senate Bill No. 1373 was filed on 6 October 2022 which seeks to protect online gig economy workers [5]. In February 2023, the House of Representatives passed House Bill No. 6718, providing protection and relief to Filipino freelance workers [6].

Early career researchers (ECRs) carry out most of scientific research and can play a critical role in advancing health policy and systems research (HPSR) by bringing new perspectives and diversity to the field [7]. Investing in the development of ECRs can help to build sustainable research capacity in LMICs [8]. Similarly, freelance workers are becoming an increasingly common presence in healthcare and research [9, 10]. With the rise of digital technologies and the availability of online tools, freelance researchers can work independently or with collaborators to conduct research and contribute to scientific knowledge.

Previous research has identified a range of challenges that freelancers and ECRs face. Freelance workers often work independently, which can be isolating, and may struggle with income stability and social protection [11]. ECRs, on the other hand, often lack mentorship and training opportunities that hinders professional development and career advancement [12]. Both groups struggle with work-life balance due to heavy workloads or irregular working hours [9, 13].

As the field of scientific research continues to evolve, both groups are likely to play an increasingly important role in advancing knowledge and making a positive impact on health systems. Existing literature has focused on the experiences of ECRs in an institutional academic setting. However, little is known about the experiences of freelance ECRs in the health policy and systems (HPS) space in LMICs. Understanding the experiences of freelance HPS ECRs can inform the development of strategies to support a new group in the next generation of HPS researchers, and promote a more inclusive and diverse research environment.

## The Alliance for Improving Health Outcomes

The Alliance for Improving Health Outcomes (AIHO) is a non-stock, non-profit public health organization in the Philippines established in 2013 [14]. Its mission is to *“enable people to make health systems work for people”.* One of the ways AIHO achieves this mission is by mentoring young professionals who participate in research, program design, policy development, or project implementation. In 2020, the organization signaled its continued commitment to its mission by including *“Focus on people by building an ecosystem for public health professionals, enthusiasts and advocates”* as one of its five-year strategic goals [15]. In 2021, at the direction of the Board of Trustees, a Mentoring Committee was created to establish the organization's mentoring framework [16]. As a non-profit, researcher salaries in AIHO are sourced completely from research grants. All members of a research team are contracted only for the duration of the grant. From 2013 to 2020, AIHO issued 797 contracts related to its research and development work, with almost 25% (198 contracts) of these for entry level and research assistant positions [15].

## Methods

This is a qualitative study that used semi-structured key informant interviews (KIIs) and focus group discussions (FGDs) to explore the experiences of freelance HPS ECRs in AIHO. The KIIs and FGDs occurred as part of management improvement efforts in the organization. Target participants were current or former freelance researchers in AIHO and were purposively sampled. Participants were contacted via email, which contained the purpose and proposed date and time of the KII or FGD. Participants confirmed their participation by confirming their attendance to the scheduled KII/FGD and voluntarily participated in these. Prior to each KII and FGD, participants were reoriented on the purpose of the data collection and verbal consent was sought.

The interview guide (Additional file 1) was developed based on relevant literature on ECRs [13, 17–19]. The guide consisted of open-ended questions to encourage participants to share their experiences as freelance HPS ECRs in the aspects of: (1) work arrangement, (2) tasks, (3) expectations from senior researchers, (4) development in the HPSR field, (5) relationship with freelance ECR peers, and (6) motivations for continuing to work as a freelance ECR. No revisions were made to the interview guide as data collection progressed.

Four FGDs with 2 to 4 participants each and 2 KIIs were conducted from 18 to 22 March 2022 through Google Meet. These were video-, audio-, and chat-recorded with the consent of participants. Each KII or FGD lasted between 60 to 90 minutes. Only the participants and interviewer (RKS) were present in the call. No repeat interviews were conducted.

The interviews were transcribed verbatim in the original language used (MAN), then translated into English during data analysis (RKS). Each transcript was reviewed for accuracy by 2 researchers (RKS and MAN). Data were analyzed using thematic and content analysis, guided by the method recommended by Creswell [20]. Thematic and content analysis use a systematic process of coding, examination of meaning, and provision of a description of the social reality through the creation of themes. Through an abductive approach, the themes and codes were created based on the topic guide developed. New themes and codes were generated as they emerged. Two researchers (RKS and MAN) coded the data using both a priori and emergent codes. Any coding conflicts were resolved through discussions until intercoder agreement was reached. Interpretation and conclusions from the data were developed by 2 researchers (RKS and MAN) with consideration for its context and relationship between themes.

## Results

Fifteen (15) current and former freelance researchers with AIHO participated in the study (Table 1). Most participants are female, under 35 years old, and with an undergraduate degree as their highest educational attainment.

**Table 1:** Characteristics of participants

Sex	
Female	10
Male	5
Age	
Under 35 years old	14
36 to 45 years old	1
Educational attainment*	
Undergraduate degree	11
Postgraduate degree	4
Medical degree	2
Years working as a freelance researcher	
< 5 years	8
> 5 years	7

*Participants with a medical degree and a postgraduate degree were counted twice under the appropriate category

A summary of the findings is presented in Table 2. The following section details the results of the study.

**Table 2:** Summary of results

Theme	Codes	Key findings
Work arrangement	Uncertainty and anxiety Valuing freelancing flexibility and opportunity	ECRs reported feeling anxiety and uncertainty that comes with being a freelance HPS ECR. The lack of a structured career path is a challenge, in contrast with the perceived stability that being an institution-based researcher provides. Working with multiple organizations provides good compensation, projects, and publications They recognize the importance of managing their time effectively to balance multiple projects, but that they heavily need to rely on performance and networks to secure future projects.
Tasks and support	Roles and responsibilities of ECRs Support and meaningful feedback Acknowledgement of potential and valued contributions	ECRs play a crucial role in supporting technical aspects of research projects, including data collection, literature review, analysis, report writing, and event coordination. Overall, the prevailing culture is that ECRs will need to be flexible and willing to take on a range of tasks to support a project's success, even if those tasks are not explicitly stated in their contract. Imposter syndrome is common among ECRs who may lack confidence with unfamiliar tasks. Personalized performance feedback is important for ECRs, as the current feedback process is limited to their outputs which may not necessarily capture the full extent of the ECR’s abilities and potential.
Expectations from and working style compatibility with leads or senior researchers	Ideal project leadership qualities Communication and commitment issues in research leadership Working style compatibility	From the perspective of freelance ECRs, effective project leadership requires a combination of technical expertise, project management skills, and strong communication skills. Lack of time commitment by leads or consultants delays projects and burdens ECRs to compensate and complete the work, leading to the ECR’s dissatisfaction. Work style compatibility is viewed by freelance ECRs as important for successful vertical relationships. ECRs feel that as young researchers, their technical skills are important, but compatibility with their leads or supervisors matters more. A mismatch with a supervisor can be detrimental to a freelance ECR’s career, since they heavily depend on supervisor referrals for future work.
Growth or development in the HPSR field	Unclear career path as a freelance ECR in HPSR Professional development, mentorship, and networking Facing the doctor-centric culture in HPSR	ECRs have to build capacity independently, since they do not have funding support usually available for institution-based researchers. Mentorship is crucial for a freelance ECR’s career growth, as mentors who believe in them, give opportunities, and understand that mistakes are part of the learning process can foster development. The prevailing doctor-centric culture in public health and HPSR is another challenge that freelance ECRs face. This culture often restricts opportunities for qualified individuals who are not medical doctors, creating a glass ceiling for non-doctors that could limit career growth.
Value of peer relationships	Peer support and relationship dynamics Peer relationship beyond work	Relationship-building in a virtual environment is challenging for freelance ECRs, but a shared interest in public health connects them. They appreciate having a support system of peers who understand their experiences, which helps to prevent burnout and maintain motivation. Having peers also helped them build nonprofessional relationships, leading to a support system that aided in alleviating stress. Freelance ECRs value having someone who understands their niche community, making it easier to discuss complex topics on public health.
Motivations to continue as an HPS ECR	Impact and contributions in HPSR Mentorship and shared values	The motivation of freelance ECRs stems from learning, growth, and the potential for impact in the public health field. Participants find HPSR work to be important and inspiring, and appreciate its empowering environment. While they acknowledge the challenges and uncertainties of being a freelance researcher in this field, they find motivation in the network and potential mentors available, as well as the desire to help those interested in public health.

### Work arrangement as a freelance ECR

#### Uncertainty and anxiety

Being a freelance HPS ECR is a fulfilling career choice, but it requires individuals to navigate uncertainties and challenges to succeed. ECRs reported feeling anxiety and uncertainty that comes with being a freelance HPS ECR. The flexibility of freelancing enables working on multiple projects, but freelance ECRs also realized they must identify research interests and not accept every offer despite job insecurity. They also acknowledged that the lack of a structured career path is a challenge, in contrast with the perceived stability that being an institution-based researcher provides.

*“You have thoughts about what you're going to do after a project. At this point, we're dependent on project leads tapping us to be part of their team.”*

*“You have to deal with this uncertainty, with that sense of directioning for yourself. At the start, there is that anxiety that I'm doing contractual work. At one point, I didn't want to say no to joining projects. In my first year working, I did 3 projects at the same time but I realized there are topics I'm not that interested in. But I didn't want to say no. I'm also scared if projects will ever be offered to me in the future.”*

#### Valuing freelancing flexibility and opportunity

Despite the challenges, ECRs generally expressed satisfaction with contracted work, and they appreciate the opportunities it provides. Working with multiple organizations provides good compensation, projects, and publications. However, they understand that the profitability of HPSR for non-traditional research institutions is limited, making the chances of eventually regularizing freelance ECRs slim. They recognize the importance of managing their time effectively to balance multiple projects, but that they heavily need to rely on performance and networks to secure future projects.

*“In every project you enter, you always have to be at your best and give it your all because you don't know when you're gonna have another project. You have to prove that you're worthy and you can do it.”*

*“You cannot rely on a single organization to give you a lot of work because there are a lot of freelance researchers. Working with multiple institutions gave me good compensation, projects, and publications. But at the end of the day, I'm not sure if it was just luck. In general, we do not have a safety net within this type of work. There's no structured way of moving forward.”*

### Tasks and support

#### Roles and responsibilities of ECRs

ECRs play a crucial role in supporting technical aspects of research projects, including data collection, literature review, analysis, report writing, and event coordination. Their tasks and responsibilities are often outlined in contracts, but they are also expected to be flexible and willing to take on ad hoc tasks, which can be a source of stress for some ECRs. Overall, the prevailing culture is that ECRs will need to be flexible and willing to take on a range of tasks to support a project's success, even if those tasks are not explicitly stated in their contract. Imposter syndrome is common among ECRs who may lack confidence with unfamiliar tasks, but building knowledge and skills through reading and learning on the job is viewed as essential.

“*As a Research Assistant, you're in charge of literature review, writing. Then further on you may decide on the data collection process and data cleaning process. Questionnaire design, setting it up, creating data collection systems, pilot testing, monitoring data collectors, and descriptive data analysis. On top of that, there are presentations or reports. Research Assistants do the bulk of the technical work. Maybe a little bit of project management work, but it would depend on the project.”*

*“I always found myself in a position that I never...literally that wasn't what was in the contract. I happened to be in projects that were morphing day by day. In that case, as an individual, you had to just morph with it and work with the team.”*

*“I want to plan and anticipate what tasks I'm going to do. I don't like ad hoc tasks because it throws off my plans and how I plan to do my work. It's also stressful because all this time we were expecting consultants to do it (the ad hoc tasks assigned) and I feel I have a capacity gap.”*

#### Support and meaningful feedback

The level of support and guidance provided to ECRs varies depending on the project and lead. Some projects provide ample support and guidance, while others rely on the assumed existing knowledge and experience of an ECR. Personalized performance feedback is important for ECRs, as the current feedback process is limited to their outputs which may not necessarily capture the full extent of the ECR’s abilities and potential. They expressed a desire for more personalized feedback on their performance and progress, which can alleviate their anxiety and improve confidence.

*“I appreciate that I'm being trained and assisted when I'm tasked to do certain deliverables. Even though I felt like my skills were inadequate, I was able to refresh my knowledge because I was taught.”*

*“Sometimes I feel my own anxiety. The things I'm doing...does it pass their standards? There's no way of knowing whether they're okay with it.”*

*“Rarely do we have a briefing about our performance and, personally, I think that has become an insecurity for me because a lot of this is on the job. Since I don't have a basis of what my performance was, I'm left to judge my own work. That's kind of anxiety-inducing for me.”*

#### Acknowledgement of potential and valued contributions

While ECR roles and responsibilities may be vague, they appreciate when project leads value their opinions and trust them to lead important tasks, showing that they are a vital member of the team. Ad hoc tasks or tasks beyond their technical capacity can be challenging but also an opportunity to showcase their potential and be valued for their skills. This acknowledgement of their potential is viewed as a double-edged sword, but ultimately appreciated.

*“There's also this sense of freedom you have when they ask you to do stuff. I think this is part of the struggle with ad hoc tasks or tasks beyond our technical capacity. I feel like the fact that that was delegated to us means that we were valued. Our potential was acknowledged. I think it's a double-edged sword and that's the good part of it. They think you are capable of doing this and that's why they trust you enough to do this even if it may not be your job.”*

### Expectations from and working style compatibility with project leads or senior researchers

#### Ideal project leadership qualities

From the perspective of freelance ECRs, effective project leadership requires a combination of technical expertise, project management skills, and strong communication skills. Project leads should have a clear understanding of the research, including its goals and objectives, and be able to provide direction and guidance to the team. Effective time management, organization, and meeting facilitation skills were also seen as important qualities in a project lead. Additionally, leads should be able to delegate tasks appropriately, monitor progress, and provide feedback to team members. Technical expertise is crucial, but project leads should have operational knowledge to ensure that tasks are executed smoothly.

*“For me, I expect that they will give the overall direction of the project. The project lead should have a clear understanding of the way the project works because they're the top in their fields. They should guide us on how to do the tasks and provide the project vision.”*

*“The bulk of technical work should be coming from them because as Research Assistants, we're just there to support them. Big decisions, methodologies, and analysis should be done by them and not us. They're the experts on that certain field so they're supposed to be the ones who impart knowledge and steer the project forward.”*

*“Know how to delegate tasks. As the lead, you should know how to make the team work efficiently. Leads should also understand the tasks they delegate. Why lead if you don't know that? Leads are answerable to what the entire project team does.”*

#### Communication and commitment issues in research leadership

Poor communication and lack of commitment from leads or consultants can have a negative impact on the project and the well-being of freelance ECRs. They expressed frustration with project leads who are too busy or unavailable, leaving them without guidance and unsure of their tasks. Lack of time commitment by leads or consultants delays projects and burdens ECRs to compensate and complete the work, leading to the ECR’s dissatisfaction. To prevent burnout of and placing unrealistic demands on ECRs, project leads must be able to comprehend the workload and time requirements for research tasks they delegate.

*“It's more related to the time they commit to the project. I feel like for some senior consultants, it's not for a lack of desire to contribute to the project, but we are engaging with very busy people. In those moments, the Research Assistants would compensate. It's not something we can negotiate.”*

*“It's really an issue of dedication and commitment. When they say they would be providing feedback by a certain time, I hope they also honor their word. Research Assistants try their best to meet deadlines of senior consultants when they ask it. It should be mutual that they also honor their word when they say they'll give feedback by this time.”*

*“I think the worst that can happen to a Research Assistant is when there's a mismatch in timelines because a lead doesn't understand how much work goes into something. ‘Get this done by tomorrow’, but do they know how much reading goes into writing a proposal?”*

*“I guess this is why a lot of Research Assistants are burned out. Research Assistants do the work when supervisors have very little idea of how to do the work. The burden of decisions goes to the Research Assistant. It's a mismatch of expectations because they expect too much from Research Assistants and too little of themselves. Why are Research Assistants always expected to be really good right off the bat? But for project leads, shouldn't we also have a high standard or set of expectations?”*

#### Working style compatibility

Work style compatibility is viewed by freelance ECRs as important for successful vertical relationships. ECRs feel that as young researchers, their technical skills are important, but compatibility with their leads or supervisors matters more. They need to identify the working style of more senior researchers, then subsequently adjust to have an effective working relationship with them. A mismatch with a supervisor can be detrimental to a freelance ECR’s career, since they heavily depend on supervisor referrals for future work.

*“Compatibility matters more than technical capacity. I'm not comfortable with buzzer beaters. I'm stressed about that. It's difficult for me if the project lead is like that, especially when Research Assistants don't have a say on how things should happen. It was hard to adjust because you need to be flexible with the working style of senior researchers. That was more stressful for me than any technical skill required for the project.”*

*“I also witnessed the impact of a supervisor and Research Assistant mismatch. I've seen this a lot, where there's been a mismatch and that became the end of things for the younger staff. They weren't given another opportunity. It's a case-to-case basis and it's unfortunate when you're mismatched with a supervisor. When that happens, that might be your end in freelancing work.*

### Growth or development in the HPSR field as a freelance ECR

#### Unclear career path as a freelance HPS ECR

Freelance ECRs view the career trajectory in HPSR as abstract and challenging. Portfolio-, reputation-, and profile-building are crucial, as the job market is not as clear-cut as in the institutional academic set up. Freelance ECRs are concerned about not being able to move up from being a Research Assistant to becoming Research Associates, Project Managers, or Project Leads despite having regular projects. They also have to build capacity independently, since they do not have funding support usually available for institution-based researchers.

*“I've been a Research Assistant for a long time. I want to hold higher positions but I'm not sure what skills are needed or what should be in my CV. I know a Research Assistant who took project management training but that's really expensive.”*

*“I don't know how to move forward. Is being a Research Assistant going to give me Masters opportunities, work opportunities? I'm optimistic about that but the path isn't super clear to me. I want to have the same influence or position close to my mentors but I don't know how to get to that level.”*

*“You have to build your capacity by yourself. In corporate, they give you funding for training opportunities. But here you're on your own.”*

#### Professional development, mentorship, and networking

Freelance ECRs seek advanced degrees or project management training to enhance their employability for higher-level positions, exploring varied skill and knowledge acquisition paths. Networking is seen as essential for career advancement to identify job openings. Mentorship is crucial for a freelance ECR’s career growth, as mentors who believe in them, give opportunities, and understand that mistakes are part of the learning process can foster development.

*“The nature of the work and being an introvert are also factors. I'm doomed because I don't know where to start. How does networking work? I also know that the referral system is beneficial because that’s how I got into this organization in the first place.”*

*“It's not just yourself but also an external person who would tell you that it's alright to make mistakes. I mean, it's alright that you will have unpleasant parts in project management or in the project and it's okay. You brush it off then that's a learning experience and that's okay. We learn again. We make mistakes again and it's okay. It takes someone to do that.”*

#### Facing the doctor-centric culture in HPSR

The prevailing doctor-centric culture in public health and HPSR is another challenge that freelance ECRs face. This culture often restricts opportunities for qualified individuals who are not medical doctors, creating a glass ceiling for non-doctors that could limit career growth. While the meritocracy model is still present, it is a doctor-centric model that perpetuates the culture. To address this issue, the participants suggest welcoming a wider pool of ECRs and consultants, and attracting talent by going outside the usual network of doctors. However, the lack of non-doctors in leadership positions could make it challenging to effect change. Despite the challenges, some participants hope for a cultural shift in public health in light of the recent COVID-19 pandemic.

*“I'm not a doctor, not even an allied health professional. I really couldn't see myself staying because there was no opportunity for someone like me, and it became frustrating.”*

*“There are senior consultants who are trying to address this issue. But at the end of the day, even if you're a project lead, you're still asked: ‘Are you a doctor?’ I know a lot of people who are studying abroad that don't want to come back because of this.”*

*“There’s definitely a glass ceiling for non-doctors. How does the doctor-centric culture affect me? I don't know, I guess I just have to come to terms with the fact that there will be a ceiling. It's up to us individually to decide what our expectations are and what we're willing to settle with if we stay here or what opportunities we'll want to take elsewhere.”*

*“No matter what you say about giving opportunities to non-doctors, if there are only a few non-doctors and you don't have anyone in a leadership position to influence, nothing will happen. And that trickles down. As long as leadership is filled with doctors, all they're going to look for are graduates of medical school.”*

### Value of peer relationships

#### Peer support and relationship dynamics

Relationship-building in a virtual environment is challenging for freelance ECRs, but a shared interest in public health connects them. They appreciate having a support system of peers who understand their experiences, which helps to prevent burnout and maintain motivation. Working with the same peer on multiple projects has been particularly beneficial for building rapport. Having someone to talk to without judgment is essential, especially when they have questions or concerns about their work or performance.

*“It's difficult to establish relationships now because since I started working, everything has been online. I haven't personally met anyone. I really prefer face-to-face interactions and while it's possible to do that, I worry about how to talk to my other teammates. In one project, I'm the sole research staff. Then in another project, we have limited interaction. There are no ‘hellos’ or ‘how are yous’. It's difficult but we just need to find ways.”*

*“Research Assistant peers are super important. In 2 projects, I worked with the same peer. It made a difference in how we did our work together that we already knew each other beforehand. There are things only Research Assistants understand so it's great that you have someone you can talk to without judgment, especially if it's a stupid question.”*

#### Peer relationship beyond work

Having peers also helped them build nonprofessional relationships, leading to a support system that aided in alleviating stress. They acknowledged that having someone to talk to about work and personal matters made them feel more comfortable.

*“We have that support system and it's valuable. It's a way to have friendships from work and decompress. It's just easier when you have someone who can relate to the things you're working on. We're a niche community. I can't tell my other friends about health financing and support value. There's so few people you can talk to about these things in a way that they can relate to and appreciate it.”*

*“It's really not easy to find those who are into public health the way we are. There are some conversations you could only have with people in this circle. I'd love to cheer on and celebrate how everybody's moving forward.”*

### Motivations to continue as an HPS ECR

#### Impact and contributions in HPSR

The motivation of freelance ECRs stems from learning, growth, and the potential for impact in the public health field. Participants find HPSR work to be important and inspiring, and appreciate its empowering environment. ECRs see the potential for their skills to contribute to policy building.

*“Personally, I didn't really consider entering the public health field at first. I didn't appreciate the field at the start. But when I started doing research, I saw how public health is applied in real life and I realized how important it is. Everyone believes that what they're doing contributes to nation-building. I was inspired by that.”*

#### Mentorship and shared values

While they acknowledge the challenges and uncertainties of being a freelance researcher in this field, they find motivation in the network and potential mentors available, as well as the desire to help those interested in public health.

*“What motivated me at that time is the network in this organization that you could not get access to anywhere else. I thought the network and the potential mentors you could get was basically unrivaled and if you are someone who wanted to work in public health policy, my perspective was there is no better place to be.”*

*“Learning from very like-minded people. It's idealistic but what really pushes me to sustain this type of work is the people I work with who also share the same values. That alone is enough to sustain me. There are times that you're stuck, you don't know what you're doing. But what anchors me sometimes is that some people really believe in you.”*

## Discussion

The COVID-19 pandemic exposed public health’s workforce needs, including gaps in young professional integration [21, 22]. Post-pandemic, there has been a push for scientific change towards intellectual humility and inclusive spaces for broader peer contributions in post-normal science [23, 24]. ECRs are the majority of the research workforce and have a stake in the research culture they will inherit as its future leaders [25]. The future of work will include a mix of full-time workers, part-time workers, and freelancers [26]. Changes in the workforce accelerated by the pandemic has also given rise to worker agency [27], which will reduce workforce voice gap on issues such as working conditions and fair treatment [28]. Despite advancement of labor policies for freelancers in the Philippines, documenting their lived experience remains a literature gap [11]. This paper contributes to global literature by offering the perspectives of HPSR practitioners at the intersect of being an ECR and freelancer in the Philippines. ECRs are less likely to feel secure in their jobs compared to more senior researchers [29, 30]. From our findings, freelance HPS ECRs experience similar challenges as institution-based ECRs, with more precarity.

Similar to our findings, existing literature has documented the sense of uncertainty that ECRs feel due to an unclear career path [31–33]. For freelancers, this is exacerbated by the fact that they do not have a stable source of income outside of a short-term contract. They continuously need to look for more work (a task classified as unpaid work), often a difficult and time-consuming process for freelancers [9, 34]. With research funding still focused on senior academics, ECRs continue to rely on the successful grant acquisition of senior researchers [12, 22]. This continuous process of looking for work may sideline efforts of freelance ECRs to focus on career progression activities due to the constant economic pressures they face.

As part of the knowledge economy, the work of researchers heavily involves problem solving [35]. This requires lifelong learning to maintain and improve their competitiveness and capacity to solve complex problems [36, 37]. But because their career path is unclear, it is difficult for ECRs to determine the skills they want or need for advancement. Their training needs will typically depend on skill gaps they identify while exploring different research areas and methodologies [36, 38]. However, performance feedback from supervisors for freelance ECRs is lacking, making gap identification difficult despite upskilling being the apparent option for career progression. Training in research also means taking specialized courses by experts or reputable institutions. This incurs high costs that burdens freelance ECRs since no institution can subsidize their training. When they do decide to invest in upskilling, they may be aware that this may not necessarily translate to career advancement. As per our findings, a freelance ECR’s employability relies on other factors such as reputation-building, referrals, and research experience.

It is common for freelancers to feel lonely and isolated because of the lack of social integration that typically happens in an office job [11, 39]. In reality, each freelancer working in isolation is working with others, often anonymously, through digital platforms [40]. This phenomenon was not observed in our interviews. Freelance HPS ECRs build relationships with other freelancers due to the collaborative nature of research, even with the challenges of working because of the COVID-19 pandemic. Peer relationships with other freelance HPS ECRs is valued because of their shared experiences in a niche community. Networks of ECRs emerge informally and naturally [41], and freelance ECRs must learn to foster these relationships as a platform for future collaboration, opportunities they usually cannot access, peer learning, and career planning [42].

Freelance ECRs in our interviews identified issues such as being assigned tasks outside their contract, heavy workloads, and compensating for project leads. These issues exist within a set research culture, the establishment of which is largely credited to research leads, managers, and supervisors [29]. While stressful and anxiety-inducing, there was an acceptance by freelance ECRs that these are just part of the job. They rationalized these experiences by reframing it as a learning opportunity since they are new to the field. Freelance ECRs continue to work in HPSR for networking, the chance to be mentored, and the importance of their work on nation-building. These motivations are even more crucial in consideration of the precarity that freelancers deal with. But the current research culture may be exploiting these motivations, a documented experience for ECRs. Exploitative practices that have been reported by ECRs include heavy workloads, long hours, being assigned difficult tasks, and being asked to do tasks for their supervisors [13, 29, 41, 43–45], most of which were also reported in our interviews. Those on short-term contracts, like freelance ECRs, are especially vulnerable. They are powerless and are unable to negotiate for a better research culture that would improve their work experience. What underpins this may be the role of power dynamics in the research culture. Freelance ECRs depend on senior researchers for job opportunities, leading to a pattern where they tolerate a supervisor’s poor performance for future benefit. Senior researchers must recognize their privileges and contributions to research culture. They define the research culture, which also means they are in the best position to shift it into a positive experience for all [29, 46].

Literature has examined why doctors need public health training [47–49], but there is limited literature on a culture that limits the role of non-medics in public health. Our interviews documented the glass ceiling that a doctor-centric culture in public health and HPSR creates for freelance ECRs. A 2018 study that explored the multidisciplinary extent of public health in 12 countries found that specialized public health training programs are limited to medics [50]. The study also found that brain drain had an unintended progressive effect—it resulted in the opening up of senior public health positions to non-medics. In some countries though, medics continue to maintain an advantage in public health and its leadership positions, similar to the experience of freelance ECRs [51–53]. The ecological model of health has led to the recognition that health is a complex issue that requires multidisciplinary solutions [54–57]. A primary example is COVID-19, where sectors of health, transport, education, and finance, among others, worked together to mitigate its impact [58–60]. In addition, the demand for collaborative and multidisciplinary research has grown from funders and policymakers, driven by a need to solve increasingly complex health problems [61]. It is clear that public health has morphed into a multidisciplinary field and profession distinct from medicine. When non-medic disciplines are marginalized in public health practice and HPSR, it is a waste of talent, a barrier to the development of a public health and research workforce, and ultimately a disservice to public health [50, 62, 63].

This study has several limitations. First, it has a small sample size of 15 participants who are or were freelancers in the same organization in the Philippines. The primary concern here is if the study reached a saturation point, which is said to be achieved in samples of 25 to 30 participants for interviews [64]. This limits the identification of more issues or challenges across different HPSR institutions in the country. Nevertheless, it is essential to contextualize this limitation within the framework of our research. Our investigation targets a distinct and underexplored demographic in both local and global contexts. The insights gained from this specific group of participants retain their validity and relevance, underpinning the significance of our findings despite the constrained sample size. Second, the FGD and KIIs were conducted virtually, and some limitations were observed in engagement specifically with ECRs that the interviewer had not met in person before COVID-19 control measures were put in place. To mitigate this, turning on video cameras was encouraged but voluntary during data collection, which aimed to supplement the lack of social cues in a virtual space and increase familiarity between the participant and interviewer [65, 66]. Finally, while efforts were made to ensure rigor of data collection and analysis, researcher bias and interpretation may still have influenced the results as both are freelance researchers.

## Conclusion and recommendations

This paper provides valuable insights into the experiences of freelance HPS ECRs in the Philippines. The findings suggest that these researchers face a range of challenges related to navigating the research landscape, exacerbated by the precarious nature of their work. Harnessing and supporting freelance ECRs is crucial to prevent multidisciplinary talent loss in HPSR amidst accelerating workforce changes and increasingly complex health issues. To better support and leverage the potential of freelance HPS ECRs, we recommend the following policy and organizational interventions:

1. Drawing on the insights from our study, there's a clear need for structured mentorship and professional development programs tailored to the unique needs of freelance ECRs. These programs should focus on enhancing research skills, grant writing, and project management capacities. Importantly, such programs should facilitate direct access to senior researchers and policymakers, fostering relationships that can guide career progression and open opportunities for impactful research.
2. We urge research funding agencies to innovate their grant-making process and models to allocate HPSR grants specific for ECR-led projects. This not only encourages the development of research and leadership skills, but also recognizes and validates the essential work conducted by these researchers.
3. The creation of a formal network or platform for Filipino HPS ECRs, similar to the Alliance Hive by the Alliance for Health Policy and Systems Research [67], can serve multiple functions such as sharing resources and opportunities for ECRs, forum for peer support, and elevating the visibility of ECR contributions to HPSR.
4. Finally, with the rising number of young freelancers in the Philippines, we urge stakeholders to advocate for policies that provide robust support for freelancers. This encompasses not only legal recognition and protection but also active inclusion of freelancers in policy-making processes, advisory boards, and strategic planning sessions across all sectors. Such efforts should aim to ensure that freelancers are recognized as integral to the Philippine economy and its development.

Future research in this topic could explore the experiences of freelance HPS ECRs in other contexts, both in the Philippines and in other LMICs. It would also be valuable to explore the perspectives of employers and senior researchers on the role of freelance HPS ECRs. Exploring the experiences of freelance ECRs who have transitioned to more established roles in public health or HPSR could provide insight into effective strategies for career development and progression. Insights can also be gathered from freelance HPS ECRs on how research funding trends (risk-aversion, short-termism, fewer but larger grants) affect them and their work.

### Supplementary Information

**Additional file 1.** Interview guide.

## Data Availability

The datasets generated and/or analyzed in this paper are available from the corresponding author on reasonable request.

## Abbreviations

AIHO: Alliance for Improving Health Outcomes
ECR: Early career researchers
FGD: Focus group discussion
HPS: Health policy and systems
HPSR: Health policy and systems research
KII: Key informant interview
LMICS: Low- and middle-income countries

## References

[1] Payoneer. The global gig-economy index. Payoneer; 2019.

[2] Payoneer. Freelancer Income Report. Payoneer; 2020.

[3] Dulay J. 2017 Philippine state of freelancing. Virtual Assistant Bootcamp 2017. https://vabootcamp.ph/blog/2017-philippine-state-of-freelancing/. Accessed Feb 26 2023.

[4] Cos W. Filipinos turn to “gig economy” for additional income. ABS-CBN News 2022. https://news.abs-cbn.com/business/08/04/22/filipinos-turn-to-gig-economy-for-additional-income. Accessed Feb 26 2023.

[5] 19th Congress - Senate Bill No. 1373 - Senate of the Philippines 2022. https://legacy.senate.gov.ph/lis/bill_res.aspx?congress=19&q=SBN-1373. Accessed 26 Feb 2023.

[6] Press and Public Affairs Bureau. House passes protection bill for over 1.5 M Pinoy freelance workers. House of Representatives Press Releases 2023. https://www.congress.gov.ph/press/details.php?pressid=12384. Accessed 26 Feb 2023.

[7] Bankston A, Davis SM, Moore E, Niziolek CA, Boudreau V (2020). Why scientific societies should involve more early-career researchers. Elife.

[8] Merritt C, Jack H, Mangezi W, Chibanda D, Abas M (2019). Positioning for success: building capacity in academic competencies for early-career researchers in sub-Saharan Africa. Glob Ment Health (Camb).

[9] Berg J, Furrer M, Harmon E, Rani U, Silberman MS. Digital labour platforms and the future of work. Towards Decent Work in the Online World Rapport de l’OIT 2018.

[10] Columbia Business School-the Eugene Lang Entrepreneurship Center (2020). Why healthcare is eating the world: the gig economy’s role in the future of work.

[11] Bajwa U, Knorr L, Di Ruggiero E, Gastaldo D, Zendel A (2018). Towards an understanding of workers in the global gig economy. Glob Migr Health Initiative..

[12] Salihu TS (2020). Challenges experienced by early career researchers in Africa. Future Sci OA.

[13] Christian K, Johnstone C, Larkins J-A, Wright W, Doran MR (2021). A survey of early-career researchers in Australia. Elife.

[14] Home. Alliance for Improving Health Outcomes 2020. https://www.aiho.org.ph/. Accessed 25 Mar 2024.

[15] Sales K, Ballesteros AJ. AIHO Annual Report 2020. Alliance for Improving Health Outcomes; 2021.

[16] Sales K. AIHO Annual Report 2021. Alliance for improving health outcomes; 2022.

[17] Hobson J, Jones G, Deane E (2005). The Research Assistant: silenced partner in Australia’s knowledge production?. J High Educ Policy Manag.

[18] Dragnich A, Smith RH (2017). The care and feeding of law student research assistants. Perspect Teach Legal Res Writing.

[19] Weeks LE, Villeneuve MA, Hutchinson S, Roger K, Versnel J, Packer T (2015). What we learned about mentoring research assistants employed in a complex, mixed-methods health study. Can J High Educ.

[20] Creswell JW, Creswell JD (2017). Research design: qualitative, quantitative, and mixed methods approaches.

[21] Krasna H, Czabanowska K, Beck A, Cushman LF, Leider JP (2021). Labour market competition for public health graduates in the United States: a comparison of workforce taxonomies with job postings before and during the COVID-19 pandemic. Int J Health Plann Manage.

[22] Wong BLH, Siepmann I, Chen TT, Fisher S, Weitzel TS, Nathan NL (2021). Rebuilding to shape a better future: the role of young professionals in the public health workforce. Hum Resour Health.

[23] Rainey S, Mormina M, Lignou S, Nguyen J, Larsson P (2021). The post-normal challenges of COVID-19: constructing effective and legitimate responses. Sci Public Policy.

[24] Naumova EN (2023). Intellectual humility in public health training, research, and practice. J Public Health Policy.

[25] Kent BA, Holman C, Amoako E, Antonietti A, Azam JM, Ballhausen H (2022). Recommendations for empowering early career researchers to improve research culture and practice. PLoS Biol.

[26] Cantrell S, Weisz K, Griffiths M, Eaton K, Poynton S, Van Durme Y, et al. Unlocking the workforce ecosystem. Deloitte Insights 2023. https://www2.deloitte.com/us/en/insights/focus/human-capital-trends/2023/contingent-talent-management.html. Accessed 4 Mar 2023

[27] Cantrell S, Weisz K, Griffiths M, Eaton K, Poynton S, Van Durme Y, et al. Harnessing worker agency. Deloitte Insights 2023. https://www2.deloitte.com/us/en/insights/focus/human-capital-trends/2023/fostering-employee-empowerment.html. Accessed 4 Mar 2023.

[28] Wixom B. 5 traits of the workforce of the future. MIT Sloan 2022. https://mitsloan.mit.edu/ideas-made-to-matter/5-traits-workforce-future. Accessed 4 Mar 2023.

[29] Wellcome. What researchers think about the culture they work. In. Wellcome; 2020.

[30] Fien S, Sahay A, Watson R, Cleary M (2022). Early career researchers: will they perish before they publish?. Nurse Author Ed.

[31] Austin AE. Expectations and experiences of aspiring and early career academics. Becoming an Academic International Perspectives 2010:18–44.

[32] Hakala J (2009). The future of the academic calling? Junior researchers in the entrepreneurial university. High Educ.

[33] Brandenburg C, Ward EC (2022). “There hasn’t been a career structure to step into”: a qualitative study on perceptions of allied health clinician researcher careers. Health Res Policy Syst.

[34] Berg J (2015). Income security in the on-demand economy: Findings and policy lessons from a survey of crowdworkers. Comp Lab L & Pol’y J.

[35] Kokt D. Competencies expected from knowledge workers in the new economy. ResearchGate 2020. https://www.researchgate.net/profile/Desere-Kokt/publication/342707926_Article_-_Competencies_of_knowledge_workers/links/5f02d3ff45851550508db2b6/Article-Competencies-of-knowledge-workers.pdf Accessed 2 Mar 2023.

[36] Bhakta D, Boeren E (2016). Training needs of early career researchers in research-intensive universities. Int J Res Dev.

[37] Muzam J (2022). The challenges of modern economy on the competencies of knowledge workers. J Knowl Econ.

[38] Lown JM. Involving Undergraduate Students in Faculty Research. Adv Consum Interest 1993.

[39] Graham M, Shaw J, Graham M, Shaw J (2017). Towards another world of gig work. Towards a Fairer Gig Economy.

[40] Turkle S (2017). Alone together: why we expect more from technology and less from each other.

[41] Speelman CP (2021). Development support of early career researchers in the Netherlands: lessons for Australia. SAGE Open.

[42] Windsor F. The importance of early career networks. Rapid Ecology 2018. https://rapidecology.com/2018/04/16/the-importance-of-early-career-networks/. Accessed 3 Mar 2023.

[43] Sutherland-Smith W, Saltmash S, Randell-Moon H. Research mentoring on the edge: Early Career Researchers and academic fringe-dwelling. In: Krause, K., Buckridge, M., Grimmer, C. and Purbrick-Illek, S., editor. Research and Development in Higher Education: Higher Education on the Edge, Higher Education Research and Development Society of Australasia; 2011.

[44] Friesenhahn I, Beaudry C (2014). The global state of young scientists.

[45] Petersen EB (2011). Staying or going?: Australian early career researchers’ narratives of academic work, exit options and coping strategies. Australian Univ Rev.

[46] Chaplin K, Price D. 7 ways to promote better research culture. World Economic Forum 2018. https://www.weforum.org/agenda/2018/09/7-ways-to-promote-better-research-culture/. Accessed 3 Mar 2023.

[47] Black E. Why isn’t learning about public health a larger part of becoming a doctor? The Conversation 2016.

[48] Rao R, Hawkins M, Ulrich T, Gatlin G, Mabry G, Mishra C (2020). The evolving role of public health in medical education. Front Public Health.

[49] Koo K, Lapp I (2014). Educating the next generation of physicians in public health: the MPH for medical students. Public Health Rep.

[50] Manyara AM, Buunaaisie C, Annett H, Bird E, Bray I, Ige J (2018). Exploring the multidisciplinary extent of public health career structures in 12 countries: an exploratory mapping. J Public Health.

[51] Dhatt R, Theobald S, Buzuzi S, Ros B, Vong S, Muraya K (2017). The role of women’s leadership and gender equity in leadership and health system strengthening. Glob Health Epidemiol Genom.

[52] Coles E. The U.S. deserves the best public health doctors. They needn’t be medical doctors. STAT 2021. https://www.statnews.com/2021/10/13/improve-public-health-remove-medical-doctor-requirement/. Accessed 3 Mar 2023.

[53] Krishnan A. Public health need not be led or delivered by doctors alone 2022. https://www.thehindu.com/opinion/op-ed/public-health-need-not-be-led-or-delivered-by-doctors-alone/article65853634.ece. Accessed 3 Mar 2023.

[54] Houeto D (2021). Why public health interventions need a multidisciplinary approach to understand and address behaviours effectively?. Prev Med Epid Public Heal.

[55] Bellmann M (2012). The interdisciplinary approach of public health. J Public Health.

[56] Berridge V (2007). Multidisciplinary public health: what sort of victory?. Public Health.

[57] Dahlgren G, Whitehead M. Policies and strategies to promote social equity in health. Institute for Futures Studies; 1991.

[58] Abreu A. Close collaboration while socially distant: a multi-sectoral response to COVID-19 in Haiti. World Bank Group 2020. https://www.worldbank.org/en/news/opinion/2020/07/17/close-collaboration-while-socially-distant-a-multi-sectoral-response-to-covid-19-in-haiti. Accessed 3 Mar 2023.

[59] Commonwealth Foundation. Strengthening multi-sectoral responses to the Covid-19 pandemic. Commonwealth Foundation 2021. https://commonwealthfoundation.com/project/strengthening-multi-sectoral-responses-to-the-covid-19-pandemic/. Accessed 3 Mar 2023.

[60] Chen Z, Cao C, Yang G (2020). Coordinated multi-sectoral efforts needed to address the COVID-19 pandemic: lessons from China and the United States. Glob Health Res Policy.

[61] Iglič H, Doreian P, Kronegger L, Ferligoj A (2017). With whom do researchers collaborate and why?. Scientometrics.

[62] McPherson K (2001). For and against: Public health does not need to be led by doctors. For BMJ.

[63] McPherson K (2000). Removing barriers to career development in public health. BMJ.

[64] Dworkin SL (2012). Sample size policy for qualitative studies using in-depth interviews. Arch Sex Behav.

[65] Axtell CM, Fleck SJ, Turner N (2004). Virtual teams: collaborating across distance. Int Rev Ind Organ Psychol.

[66] Sales RK, Oraño J, Estanislao RD, Ballesteros AJ, Gomez MIF (2021). Research priority-setting for human, plant, and animal virology: an online experience for the Virology Institute of the Philippines. Health Res Policy Syst.

[67] Home. The Alliance Hive n.d. https://hive.ahpsr.org/. Accessed 27 Mar 2024.

