# Supplementary material for: Early career researchers in health policy and systems research: insights from freelancers in a non-profit organization in the Philippines
Source: Health Res Policy Syst. 2024 Apr 29;22:54. doi: 10.1186/s12961-024-01142-6 (PMC11059717; doi:10.1186/s12961-024-01142-6)
Supplement: Supplementary file 1 — Additional file 1. Interview guide. [file 12961_2024_1142_MOESM1_ESM.pdf]

1. Across different projects, what tasks have been assigned to you as a Research Assistant?
    a. Did you know that these tasks would be assigned to you prior to getting the position?
        i. How? (through what channel eg TOR, informal discussions, etc.)
        ii. Were there tasks assigned ad hoc/as needed?
            1. How do you feel about ad hoc/as needed tasks?

    b. Were these tasks appropriate for your skill set/skill level at the time?
        i. What support was provided to you so you could successfully accomplish your tasks?
            1. How were you trained/mentored?
            2. Were there feedback mechanisms present to comment on your performance or output?
            3. What were the best and improvement points for how you are mentored within projects?
        ii. What factors enabled you to accomplish your tasks?

2. In a project, what are your usual expectations from senior team members in terms of 1) their contribution to the project and 2) as a senior team member?
    a. Are there explicit discussions on the
        i. role and responsibilities of each team member?
        ii. Your expectations from each other?
    b. Do you find clarity in roles, responsibilities, and expectations useful? Why?
    c. Were these expectations met?
        i. What were specific qualities in senior team members that you think enabled them to meet your expectations?
        ii. What do you think was lacking in senior team members that resulted in them not meeting expectations?

3. What motivates you to be an RA? What do you like in your job that makes you want to continue doing it?
    a. What would incentivize you to grow/develop/in general be better in this field?
    b. What are barriers to growth/development in this field?

4. In each project, do you feel like you were an integral part of the team?
    a. How did the project or team make you feel important?
    b. Why not?

5. How often do you talk to your peers?
    a. What value do these conversations provide?
